# Home based early intervention with CareToy-Revised in infants with early brain injury: a randomized controlled trial

**DOI:** 10.3389/fmed.2026.1830574

**Published:** 2026-08-12

**Authors:** Elena Beani, Riccardo Rizzi, Veronica Barzacchi, Valentina Menici, Carolina Fioroni Ribeiro da Silva, Giada Martini, Elisa Sicola, Carlo Dani, Alessandra Cecchi, Maria Luce Cioni, Filomena Paternoster, Patrizio Fiorini, Marco Di Galante, Ugo Faraguna, Paolo Dario, Francesca Cecchi, Irene Mannari, Martina Maselli, Matteo Giampietri, Paolo Ghirri, Nevio Dubbini, Giovanni Cioni, Giuseppina Sgandurra

**Affiliations:** 1 Department of Developmental Neuroscience, IRCCS Fondazione Stella Maris, Pisa, Italy; 2 Department of Clinical and Experimental Medicine, University of Pisa, Pisa, Italy; 3 Department of Neuroscience, Psychology, Drug Research, and Child Health (NEUROFARBA), University of Florence, Florence, Italy; 4 Institute of Medical Sociology, Center for Health and Society, Medical Faculty and University Hospital Duesseldorf, Heinrich-Heine-University, Duesseldorf, Germany; 5 Department of General Pediatrics, Neonatology, and Pediatric Cardiology, University Hospital Duesseldorf, Heinrich-Heine-University, Duesseldorf, Germany; 6 Division of Neonatology, Careggi University Hospital, University of Florence, Florence, Italy; 7 Neonatal Intensive Care Unit, Children’s Hospital A. Meyer IRCCS, Florence, Italy; 8 Sleepacta srl, Pisa, Italy; 9 Department of Translational Research and of New Surgical and Medical Technologies, University of Pisa, Pisa, Italy; 10 The BioRobotics Institute, Scuola Superiore Sant’Anna, Pisa, Italy; 11 Department of Excellence in Robotics and AI, Scuola Superiore Sant’Anna, Pisa, Italy; 12 Interdisciplinary Center in Sustainability and Climate, Scuola Superiore Sant’Anna, Pisa, Italy; 13 Division of Neonatology and NICU, Department of Clinical and Experimental Medicine, University of Pisa, Pisa, Italy; 14 Department of Civilisations and Forms of Knowledge, University of Pisa, Pisa, Italy

**Keywords:** bioengineering, cerebral palsy, early intervention, infant, information technology, massage, neurodevelopmental disorders, randomized clinical trial

## Abstract

**Introduction:**

Intensive, family-centered early intervention (EI) may support neurodevelopment during a period of high neuroplasticity in infants with perinatal brain injury who are at high risk of cerebral palsy (CP). CareToy-Revised (CT-R) is a smart, home-based, parent-delivered system that provides individualized, goal-directed EI. Aims: To determine the effects of CT-R on early motor development of infants with brain injury, thus at high risk of developing CP, comparing it with another known EI, namely the Infant Massage (IM).

**Methods:**

Thirty-eight infants with perinatal brain injury, at high risk of cerebral palsy (CT-R *n* = 19; IM *n* = 19) participated in this randomized and evaluator-blinded clinical trial (ClinicalTrials.gov: NCT03211533; NCT03234959). Early motor development (primary outcome) was assessed using Infant Motor Profile (IMP) at baseline (T0) and post-intervention, immediately after the EI (T1), after 2 months (T2) and at 18 months of corrected age (T3). Secondary outcomes included assessments by Peabody Developmental Motor Scales, Second Edition (PDMS-2) and Teller Acuity Cards (TAC) at all time points and Bayley-III cognitive scale BSID-III at T0 and T3. Between-group differences at each time point were tested using the Mann–Whitney test, and longitudinal effects were evaluated using linear mixed-effects models adjusted for covariates.

**Results:**

The groups presented no significant differences at baseline. At T1, the CT-R group showed significantly higher IMP raw scores for total score, symmetry, and performance than the IM group. For PDMS-2, TAC, and BSID-III cognition, no significant between-group differences were found. After adjustment for covariates, CT-R showed effectiveness at T1 across the IMP domains (total *β* = 5.506, 95% CI 4.23–6.78; variation *β* = 6.329, 4.24–8.42; symmetry *β* = 6.075, 3.79–8.36; performance *β* = 5.657, 3.49–7.82). At T2, effectiveness remained for the IMP total score (*β* = 4.020, 2.76–5.28) and variation (*β* = 5.503, 3.43–7.57). No significant Group × Time effects were observed at T3.

**Discussion:**

CT-R significantly improved the early motor development of infants with early brain injury, supporting the potential value of intensive, individualized, home-based, and parent-delivered EI.

**Clinical Trial Registration:**

NCT03234959 on ClinicalTrials.gov.

## 1 Introduction

Cerebral Palsy (CP), according to the updated description published in 2026, is an early-onset, lifelong neurodevelopmental condition characterized by limitations in activity due to impairments in development of movement and posture (1). CP is the most common motor disability in childhood. Recent population-based studies have reported a prevalence ranging from 1.6 to 3 per 1,000 live births, with higher estimates of about 3.4 per 1,000 in low- and middle-income countries (2). CP results from early maldevelopment or injury to the fetal or infant brain. These disturbances include brain dysplasia, often linked to genetic, metabolic, or developmental processes, or acquired injury such as hypoxic ischemic events, inflammation, periventricular white matter injury, stroke, or kernicterus (3). Early identification of infants at high risk of CP relies on standardized neurodevelopmental assessments, including clinical neurological examination (Hammersmith Infant Neurological Examination, HINE) and structured tools such as the General Movements Assessment (GMA), often combined with brain MRI, which together provide high predictive accuracy in the first months of life (4). Evidence further suggests that assessments of the quality of motor behaviour provide more sensitive information on early brain functioning than traditional neurological evaluation (5). In this framework, the Infant Motor Profile (IMP) is a video-based assessment of motor behaviour, developed within the Neuronal Group Selection Theory and focused on the qualitative aspects of movement (6, 7). During the first 1,000 days of life, a sensitive period of development, the central nervous system is particularly vulnerable to insults, and, at the same time, it shows the highest neuroplastic potential for recovery (8). Infants are in an intensive process of neurogenesis, migration, synaptogenesis, axonal and dendritic growth and therefore able to receive, process, and respond to extrinsic stimuli (9). For this reason, it is important to develop evidence-based rehabilitation protocols that take advantage of this window of opportunity to maximize functional outcomes and minimize complications. Recently published guidelines based on comprehensive reviews have set the cornerstones of the Early Intervention (EI) in infants at high risk of CP (10, 11). To be effective, the EI ingredients should be early, intensive, goal-directed and based on the principles of the enriched environment (11, 12), and family-centered (13). Family-centered therapy is an approach in pediatric rehabilitation that recognizes family members not only to provide primary support to children, but also their influence on therapy adherence and outcomes (14). It focuses on understanding each family’s routines, priorities, and strengths, and builds intervention plans that fit naturally into daily life. Rather than targeting impairments alone, family-centered therapy aims to promote the child’s activity and participation within their home and community, while ensuring that parents remain informed, involved, and supported throughout the rehabilitation process (15). In addition, a recent study summarized seven elements that are important for promoting motor and overall development enhancing behavioural and neural outcomes, the E-words, earlier, engagement, exploration, enriched environments, experiences, everyday, and exercise (16). Moreover, the intervention should be multi-axial (focused on different aspects of motor, cognitive, and visual development).

Based on this approach, the CareToy system (CT) (17, 18), is conceptualized as a home-based, family-centered, goal-directed early intervention designed for infants with neurodevelopmental risk, aimed at operationalizing the key principles of evidence-based Early Intervention. It does not represent merely a technological device, but rather a structured intervention model grounded in active learning, enriched environmental exposure, and parent-mediated rehabilitation. It is designed to promote active infant participation and self-generated exploration through individualized and developmentally appropriate activities targeting motor, visual, cognitive, and exploratory domains (18–20).

CareToy system, in its original first version, has been initially used with infants at low risk for CP in a 4-week training which showed a significant improvement in visual and motor development in preterm infants without brain injury (17, 21). Based on these promising findings, the previous version of CT was updated and adapted to meet the needs of other clinical infant populations. The transition from the original CareToy (CT) system to CareToy-Revised (CT-R) represented a population-specific adaptation rather than the development of a completely new intervention. While maintaining the core principles of the original CareToy approach—including intensive, home-based, goal-directed, enriched-environment, and family-centered early intervention—CT-R was specifically redesigned to address the developmental characteristics and functional needs of infants with neurodevelopmental disabilities and those at high risk of CP. In particular, the adaptations were intended to increase opportunities for active participation, exploration, and learning despite early motor limitations, while preserving the individualized and parent-mediated nature of the intervention. Therefore, CT-R should be considered an evolution of the original CareToy approach specifically tailored for infants at high risk of CP, whose effectiveness requires direct evaluation in this clinical population. Specifically, a new modular postural system was introduced, consisting of a set of soft, velcro-based elements designed to support different safe positioning of the infant (supine, prone, sitting, and side-lying) (22).

In addition, new goal-directed activities were developed by revising the activity library in terms of images and video content, the intensity and duration of lights and sounds, the duration of the activities, and the variation in positioning. Thanks to this adaptation, the CareToy-Revised (CT-R) system has been used in a small sample of infants with Down syndrome (23). The feasibility of the CT-R training, acceptability, usability, and adherence has been demonstrated in infants at high risk of CP, supporting the implementation of this intervention in this population (24).

The primary objective of this randomized clinical trial (RCT) is to determine the effects of CT-R on neurodevelopment of infants with brain injury, thus at high risk of developing CP, comparing it with another widely known EI, namely the Infant Massage (IM). IM was selected because it is a recognized EI strategy that has shown positive effects on neurodevelopmental outcomes in infants at risk (25). This design allowed us to evaluate the effectiveness of CT-R against an established intervention already used in clinical practice.

## 2 Materials and methods

### 2.1 Study design, ethics approval and registration

This study is a randomized and evaluator-blinded clinical trial (CareToy-R RCT study), conducted and reported in accordance with the CONSORT guidelines (26). It was approved by the Tuscan Region Paediatric Ethics Committee, Italy (Ref. 84/2017). All procedures were in accordance with the Declaration of Helsinki. In addition, CT-R, as the previous CT, has been classified as a medical device without an EC mark, so an approval of a new clinical trial was requested and obtained by the Italian Ministry of Health. The study was registered on ClinicalTrials.gov (NCT03211533; NCT03234959). Further details are published in its protocol (22), and no deviations or changes were made from the published protocol.

All parents or legal guardians of participating infants provided written informed consent. In line with the approved protocol, a first consent covered the observational phase (neurological assessments and magnetic resonance image [MRI]), and a second consent was obtained before the infants enroll in the intervention phase.

### 2.2 Participants and recruitment

Infants were recruited between September 2017 and June 2020 from three Neonatal Intensive Care Units (NICUs) in Tuscany, Italy (Santa Chiara University Hospital, Pisa; Meyer Children’s Hospital, Florence; and Careggi University Hospital, Florence). A pre-eligibility screening was performed by neonatal staff before the NICUs discharge. Eligible conditions included persistent periventricular hyper-echogenicity on ultrasound, cerebral hemorrhage, periventricular leukomalacia (PVL), cerebral stroke, or a history of moderate to severe asphyxia. After, families expressing interest received written information about the project. Recruitment occurred within the infant’s first year of life and according to specific developmental milestone, given the requirements of the intervention. Parents who agreed to participate provided written consent first for the observational phase and, if eligible, later for the intervention phase.

At around 3 months post-term age, infants underwent a follow-up visit in which eligibility for the intervention phase was re-assessed using the Prechtl General Movements Assessment (GMA) (27, 28) and the Hammersmith Infant Neurological Examination (HINE) (29). Infants were included in the intervention phase if they showed persistence of abnormal general movements, absent or abnormal fidgety movements in GMA, and persistence of abnormal/specific signs according to HINE. In addition to the inclusion criteria, the start of the intervention required meeting specific motor conditions based on the gross motor section of the Ages & Stages Questionnaire, Third Edition (ASQ-3) (30). In detail, the intervention could begin only when infants had achieved initial head control but before full independent sitting position, expressed in the following score thresholds: ≥10 for ages 3–4 months, ≥5 but <50 for ages 5–6 months, and ≥10 but <30 for ages 7–8 months. Infants were monitored until these milestones were reached, and treatment could start immediately or when specific goals were set (22). Once eligibility was confirmed, written informed consent for the participation in the intervention phase was obtained from parents or legal guardians, as further detailed in the protocol (22).

The exclusion criteria were infants with polymalformative syndromes, cerebral malformations, or severe sensory impairments (retinopathy of prematurity stage III-IV-V or deafness) due to their heterogeneous etiologies and developmental trajectories, which differ from those of infants with cerebral palsy. Also, severe sensory impairments were also excluded because the CareToy-R intervention is based on multisensory stimulation (visual and auditory), which may not be adequately perceived in these cases.

#### 2.2.1 Sample size calculation

Sample size calculation was based on estimates obtained from a pilot study and of previously conducted RCT in preterm infants without perinatal brain injury (17, 19, 21). Considering an 80% power, a significant level of 0.05 and an effect size of at least 0.6, a minimum sample of 38 infants was required. To facilitate parental work and participation in the project, eligible twins were allocated into the same group.

### 2.3 Procedures

#### 2.3.1 Assessments

The assessments were performed at four time points: at baseline (T0), corresponding to the beginning of the study before the start of the intervention with CT-R or IM; immediately after finishing the intervention phase (T1); at follow-up 2 months after T1 (T2); and a final long-term follow-up at 18 months of age (T3). All assessments were carried out at the infant’s home by trained therapists and a child neurologist. At each timepoint, standardized clinical measures were administered according to the protocol (Infant Motor Profile [IMP] (6, 7, 31, 32), Peabody Developmental Motor Scales, Second Edition [PDMS-2] (33), Teller Acuity Cards [TAC] (34, 35) at all timepoints, and Bayley Scales of Infant Development III Edition [BSID-III] (36, 37) cognitive scale at T0 and T3). Each assessment was video recorded so that it could be scored offline by blinded assessors. During the whole experimental period, participation in other forms of intervention as required by the local health care providers (the standard of care) was continued. Parents of infants allocated in both groups were asked to document in specific pre-compiled diaries the main activities carried out by their infants, such as the training (CT-R/IM) duration and details, and other concomitant standard care (e.g., physiotherapy and medical visits) and any difficulties or potential adverse events related to the intervention.

Primary and secondary outcome measures

- The primary outcome was early motor development at T1, it was measured by the IMP, a video-based assessment of motor behaviour which is designed to evaluate motor abilities, variation, fluency and symmetry of the infant movement repertoire. The IMP consists of 80 items organized into the following domains: variation (25 items that reflect how broad the infant’s movement repertoire is); adaptability (15 items, assessed only from 6 months onward, that reflect the ability to select motor strategies appropriate to the task and environment); symmetry (10 items that reflect whether movements are balanced between both sides of the body); fluency (7 items that reflect the smoothness and coordination of movements); and performance (23 items that reflect the achievement of gross and fine motor milestones). The total IMP score combines all domains and provides an indicator of early motor development (6, 31, 38). As required by the IMP protocol, the IMP adaptability domain was not assessed in infants of less than 6 months (7, 38). The IMP has been validated as a discriminative measure in infants with neurodevelopmental risk, and previous studies demonstrated a solid construct validity and predictive validity for CP and other neurodevelopmental disorders. Specifically, IMP total score at 5 months shows high diagnostic performance, with sensitivity of 93% and specificity of 81% for detecting neurodevelopmental disorders, for instance, CP, with 0.92–0.95 of AUC in ROC analyses (32). IMP results are expressed in raw scores.
- Secondary outcome measures included motor functions measured by PDMS-2 (33), cognitive development assessed with the cognitive scale of the BSID-III (36, 37), and visual acuity measured by TAC (34, 35).
- The PDMS-2 is a standardized assessment of motor development for children from birth to 5 years. It is organized into six subtests: Reflexes (8 items that assess automatic postural and locomotor responses to external stimuli in infants under 12 months); Stationary (30 items that assess the ability to control body posture within the center of gravity and maintain balance); Locomotion (89 items that assess how the child moves from one place to another, such as crawling, walking, running and stair climbing); Object manipulation (24 items that assess ball skills such as throwing, catching and kicking, administered from 12 months onward); Grasping (items that assess how the child uses the hands to hold and manipulate objects); and Visual-motor integration (items that assess how well the child coordinates vision and hand movements for tasks such as building, copying shapes or cutting). Each item is scored on a 3-point scale (0–2), and subtest raw scores are converted to age equivalent scores, percentiles, standard scores and motor quotients based on normative data (33). The PDMS-2 demonstrated internal consistency of 0.76–0.999, test–retest reliability of 0.71–0.99, and interrater reliability of 0.758–1.000 (39).
- Cognitive scale of the BSID-III is a standardized assessment of early cognitive functioning for children aged 1 to 42 months. It is administered by structured, and playful tasks that evaluate non-verbal cognitive abilities, including sensorimotor development, object permanence, attention and memory, problem-solving, and concept formation. Tasks are presented in order of increasing difficulty and are discontinued once the child reaches a ceiling of consecutive failures, producing a raw score, also a percentile rank based on a normative data (36). The cognitive scale of the BSID-III has interrater reliability coefficients higher than 0.85 (40), sensitivity of 86.4%, and specificity of 94.1% for identifying children with BSID-II MDI scores below 70 (41).
- The TAC is a preferential looking tool used to assess visual acuity in infants and young children who cannot name letters or pictures. Each card has black-and-white stripes (a grating) on one side and a plain gray area on the other side. During the test, the examiner presents one card at a time and watches the child through a small peephole in the center of the card to see whether the child looks toward the stripes. The cards are shown in order from wide stripes (easy to see) to very thin stripes (more difficult to see). The smallest stripe width that still produces a clear looking response is taken as the child’s acuity. The procedure is quick (usually a few minutes for each eye), can be used binocularly and monocularly, and is feasible in clinical and research settings, including children with visual or developmental disorders (34, 35).

#### 2.3.2 Randomization

After the enrolment, a random allocation to the CT-R or the IM arm was done by the use of an automatic generation of 1:1 sets and no additional restrictions. Allocation concealment was ensured through the use of sealed, numbered envelopes prepared and handled by an independent third party, i.e., a clinician who was blind to all clinical aspects of the trial and not involved in participant recruitment or assessment. The envelopes were opened only after the infant met the motor requirements and was enrolled. No stratification was used.

#### 2.3.3 Blinding

Parents, therapists and clinical staff were aware the allocation group of infants. The scoring of the assessments was performed offline at the end of the study by blinded evaluators to the study group.

#### 2.3.4 CareToy-Revised (CT-R) early intervention (EI)

CT-R is a technological tele-rehabilitative tool which embeds several sensorized modules providing a home-based, parent-delivered and intensive EI for infants with neurodevelopmental risk (17, 18). It is inspired by a general configuration of infant play gyms (specifically, a play mat with an overhead arch and hanging toys), however, it is entirely composed of biomechatronic toys with different shapes, interactive walls with embedded lights and buttons, an arch with lights, a screen module for the promotion of visual attention and gaze, a sensorized mat and a set of wearable sensors for the infant. The system also includes a set of cameras and a laptop with telerehabilitation architecture, allowing remote connection with the clinical rehabilitative staff, in terms of download of customized daily activities and upload of data enabling remote monitoring by clinicians and the provision of highly customized training that parents actively play with their infant. Detailed, technical specifications and functioning have been previously described (18–20). For each participant in the CT-R group, a CT-R system was delivered at home. After installation, a dedicated therapist trained parents to use the system and play with their infant with CT-R during the first 5 days and then once a week for the following weeks remote videocalls, in addition to extemporary contacts or home visits if needed. The CT-R program consisted of individualized, goal-directed activities (named “scenarios”), lasting from 2 to 10 min each, organized into daily sessions corresponding to a planned total dose from 20 to 40 h across the 8-week intervention. Training frequency was fixed at 5 days per week, and parents were instructed to complete the sessions during the infant’s active and alert states, having the possibility to organize the training in the best number of sessions. Daily sessions were composed of different scenarios involving the activation of different CT-R modules and varying the initial positioning of the infant. Thanks to the telerehabilitation architecture, supported by the provision of a portable Wi-Fi router in cases where an internet connection was unavailable, data were transmitted to the clinical center and new training scenarios were downloaded. Clinicians remotely monitored the tailoring procedures and were able to modify the number of scenarios, their duration, difficulty, and other features on a weekly basis, according to the infant’s motor performance, engagement, tolerance, and parental feedback. All concomitant standard care provided by local health services was allowed and recorded. Treatment fidelity was monitored by the clinicians, data and video recordings and weekly contacts that allowed therapists to verify adherence, document adverse effects and support correct execution. Additional support from clinicians, with technical support from bioengineers when needed, was provided on demand. Through telemonitoring of the infant’s behaviour and performance, activities were tailored to the infant’s specific characteristics and needs, supporting both infants and their families. This personalized approach supported family engagement, increased parents’ confidence, and improved satisfaction with the intervention. Further details on CT-R training have been published elsewhere (22, 24, 42).

#### 2.3.5 Infant massage (IM)

For the infants allocated to the IM group, a trained therapist provided the families with a practical course of five sessions of 1 hour each. During the home sessions, she instructed the parents to properly stimulate and interact with their infant according to the sequence of movements of the IM (43). Parents were then asked to perform the IM at least 20 min a day for a minimum of 5 days per week, over the 8-week period. Parents received illustrated material describing the standard IM sequence, and the therapist encouraged tailoring the IM according to infant cues (e.g., adjusting pressure, shortening sequences, pausing when signs of fatigue or irritability were present). The IM daily diary and Infant Massage Questionnaire Parent-Infant Experiences (IMQPE) (25) were used as in CT-R group to document adherence, information on parents’ perceptions of feasibility, acceptability, and any adaptations during the massage (22). IM and IMQPE were developed to systematically monitor home-based intervention and to evaluate its feasibility, acceptability, and usability from the parents’ perspective, as are published in its protocol and feasibility study (22, 25).

In the IM daily diary, parents recorded each massage session, including the date, start and end time, total duration in minutes, and the exact sequence of movements applied. They were also asked to indicate which body regions were massaged (legs and feet, arms and hands, stomach, chest, face, and back) and to report any adaptations to the standard sequence, such as shortening, skipping, or pausing sequences in response to infant reactions like irritability, tiredness or hunger. These diaries were reviewed regularly by the therapist, who provided feedback to support correct execution of IM and to ensure fidelity to the protocol (22, 25).

#### 2.3.6 Covariates

Severity of brain injury is a well-known predictor of responsiveness to intervention. For this reason, brain MRI scans were independently assessed by a pediatric neurologist to develop a severity score based on the best available literature and consistent with previous similar trials (44–47). In case of multiple available scans, we decided to choose the series closest to term equivalent age (TEA) for preterm infants and the series closest to day 7 for infants with hypoxic–ischemic encephalopathy. MRI severity was coded as a three-level variable (Neuroimaging: 1 = likely to have mild CP; 2 = likely to have moderate CP; 3 = likely to have severe CP).

Clinical outcome was included as two covariates: presence of CP (0 = no CP; 1 = CP) and atypical motor outcome (0 = typical; 1 = atypical). Laterality of motor impairment was coded as unilateral = 0 vs. bilateral involvement = 1. Age at baseline was included as a continuous variable. The group was coded as 0 = IM and 1 = CT-R, and time was entered as categorical levels (T0–T3). In addition, the estimation of the time interaction was also calculated (group × time).

### 2.4 Statistical analysis

Firstly, descriptive analyses were performed to characterize the sample at baseline, including socio-demographic variables, clinical characteristics and all outcome measures (IMP domains and total score; PDMS total raw score; Bayley-III cognitive score). Metric variables were expressed as means (M) with standard deviations (SD), and categorical variables were reported as absolute (n) and relative frequencies (%).

The data distribution and test assumptions were examined. Normality was assessed using the Shapiro–Wilk test and visual inspection of histograms. Differences between the groups regarding IMP score, CT-R versus IM, were descriptively analyzed by Mann–Whitney tests, given the non-normal distribution of the data. Baseline differences between the CT-R and IM groups were assessed at T0. In addition, IMP delta scores were computed for each assessment interval (T0–T1, T1–T2, T2–T3) and compared between groups also using Mann–Whitney tests. The effects size was interpreted as small (<0.50), medium (0.50 and 0.80), or large (>0.80) (48, 49).

Finally, to evaluate the time interaction and if the IMP scores differed over time between CT-R and IM, we performed linear mixed-effects models adjusting it by the covariates, our primary analysis. Covariates were included based on clinical relevance: no CP vs. CP diagnosis, typical vs. atypical outcome, laterality (unilateral vs. bilateral involvement), MRI severity (grade from 1 to 3), and age. The fixed effects included time (T0, T1, T2, T3), group (IM vs. CT-R), and the interaction time × group. Multicollinearity among the fixed-effect covariates was assessed using variance inflation factors. A random intercept for infant ID was included to account for repeated measurements over time. We report the estimated coefficients (*β*), standard errors (SE), 95% confidence intervals (95% CI), *t*-test statistics (*t*-test), and *p*-values adjusted for multiple comparisons using the Bonferroni method. Distribution and model fit were examined using diagnostic *Q*–*Q* plots of residuals and *R*-squared values, respectively. Post-hoc contrasts with Bonferroni adjustment were performed as exploratory analyses to describe the observed changes across assessment time points. All analysis were conducted with R (version 4.3.1, 2023–06).

## 3 Results

### 3.1 Participant flow and baseline characteristics

**Figure 1:**
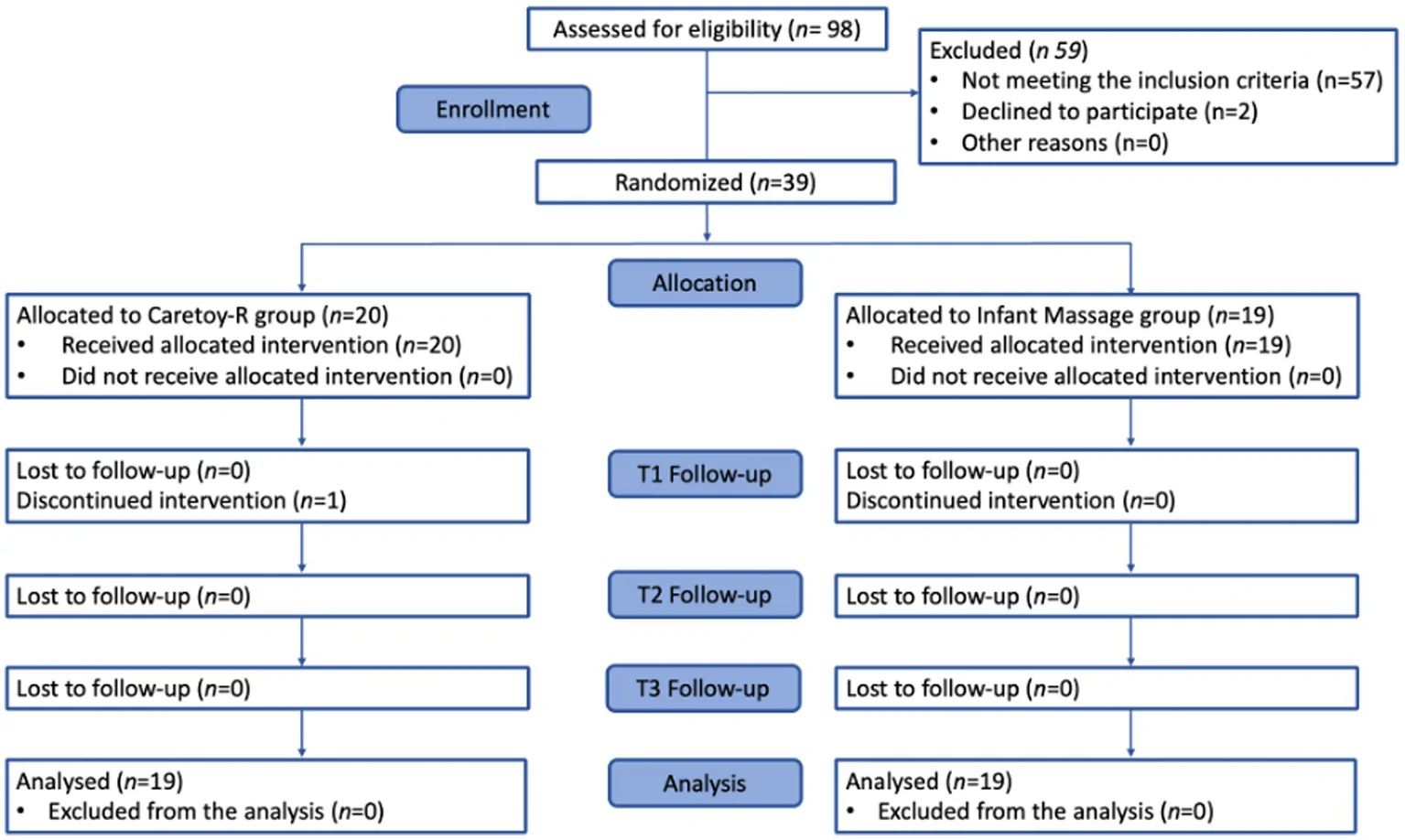
Flow of the participants through trial. n, absolute frequency; CT-R, CareToy-Revised; IM, infant massage; T0, baseline assessment before the start of the intervention; T1, end of the 8-week intervention period (primary endpoint); T2, follow-up 2 months after T1; T3, follow-up at 18 months of age.

The flowchart illustrating participant progression through the trial is presented in Figure 1. Among the recruited participants, one infant allocated to the CT-R group was excluded prior to the T1 assessment because the infant achieved independent crawling milestone, which no longer met the study inclusion criteria for the intervention.

Baseline characteristics of the study sample are reported in Table 1. Groups were equivalent at baseline for all infant characteristics except for a minor difference in age of enrollment where the IM group started the intervention at a slightly and non-significant earlier age than the CT-R group (*p* = 0.12). Also, the fluency domain scores of the IMP were slightly higher in the IM group than in the CT-R group (Table 1). The CT-R and IM were delivered as planned, and no deviations occurred that could affect the study outcomes.

**Table 1:** Characteristics of participants at baseline (T0).

Item	Sub-item	CareToy-R *n* = 19	Infant massage *n* = 19
Age at enrollment	Corrected age (months) at T0, mean (SD)	5.93 (1.99)	4.86 (1.27)
Sex	Male, *n* (%)	11 (58)	10 (53)
Gestational age	Birth gestational age (weeks), mean (SD)	35.00 (5.84)	32.37 (5.26)
	28 weeks, *n* (%)	4 (21)	4 (21)
	28–31 weeks, *n* (%)	1 (5)	6 (32)
	32–36 weeks, *n* (%)	3 (16)	3 (16)
	>36 weeks, *n* (%)	11 (58)	6 (32)
Neuroimaging at tea	Severity score		
	1 - Mild, *n* (%)	6 (32)	5 (26)
	2 - Moderate, *n* (%)	5 (26)	6 (32)
	3 - Severe, *n* (%)	8 (42)	8 (42)
	Unilateral, *n* (%)	11 (58)	7 (37)
	Bilateral, *n* (%)	8 (42)	12 (63)
Clinical measures			
IMP total score	Total raw score (SD)	64.9 (6.0)	67.3 (6.9)
IMP variation	Total raw score (SD)	62.8 (6.4)	64.8 (8.5)
IMP symmetry	Total raw score (SD)	81.8 (13.2)	83.5 (13.7)
IMP fluency	Total raw score (SD)	70.2 (8.0)	73.6 (3.9)*
IMP performance	Total raw score (SD)	48.2 (8.5)	49.1 (9.0)
PDMS total raw score	Total raw score (SD)	70.4 (25.1)	61.8 (22.6)
TAC	Cycles/degree (SD)	4.5 (2.6)	3.0 (1.8)
Bayley-III (cognitive)	Composite cognitive score (SD)	84.0 (18.3)	84.2 (18.9)

*p < 0.05.

CT-R, CareToy-Revised; SD, standard deviations; *n*, absolute frequency; %, relative frequency; T0, baseline assessment before the start of the intervention; TEA, term equivalent age; IMP, Infant Motor profile; PDMS-2; Peabody Developmental Motor Scale 2; TAC, Teller Acuity Cards; BSID-III, Bayley Scale of Infant Development-III.

The study sample included 38 infants, 19 per group. The mean age at T0 was 5.40 ± 1.74; at this time point, 4 infants in the IM group and 7 infants in the CT group were older than 6 months. At T1, the mean age was 7.62 ± 1.82 months, with 13 infants in the IM group and 18 infants in the CT group being older than 6 months. At T2, the mean age was 9.63 ± 1.80 months, and all infants were older than 6 months (Table 1).

Regarding the dose intervention, in both groups, high adherence to treatment was found with no significant difference in total dose of intervention. For the CT-R group, infants received a mean of 22.40 (SD 5.15) hours of total intervention with a mean duration of daily activities of 30.30 (SD 4.42) minutes. Regarding the IM group, participants received 21.04 (SD 8.49) hours of total treatment with a mean daily duration of 27.79 (SD 7.88) minutes. Details about family adherence to both treatments have been previously published (24, 25). No significant correlations were found between motor outcome at T1 and total dose of intervention (*R* = −0.07).

### 3.2 Clinical outcomes

**Figure 2:**
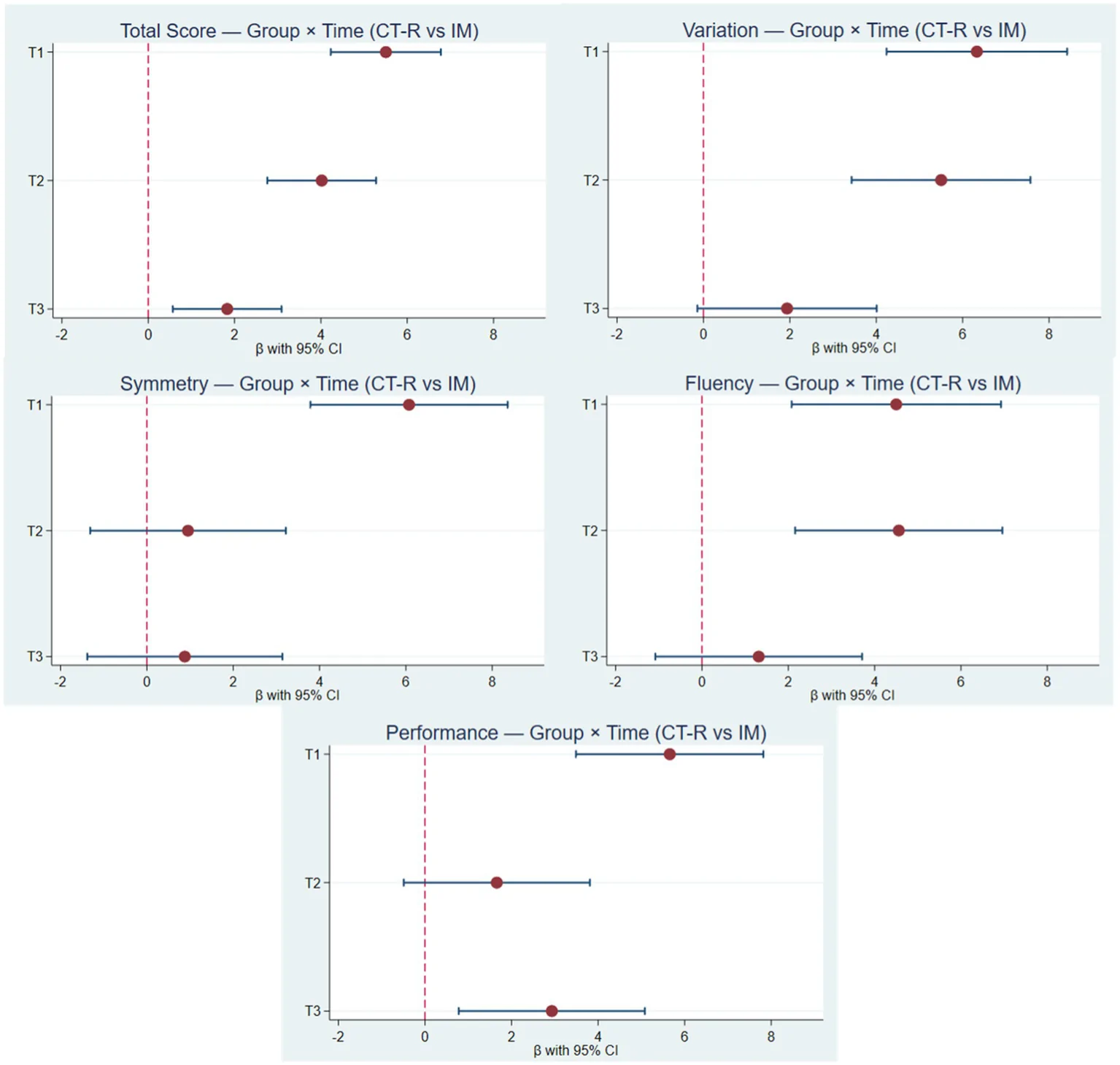
Treatment effects of CareToy-Revised versus Infant Massage on Infant Motor Profile domains over time (linear mixed-effects models). CT-R, CareToy-Revised; and IM, infant massage; T1, end of the 8-week intervention period (primary endpoint); T2, follow-up 2 months after T1; T3, follow-up at 18 months of age; *β*, fixed-effect coefficients from linear mixed-effects models for Infant Motor Profile (IMP) raw scores; SE, standard error; 95% CI, 95% confidence interval; Circles represent Group × Time treatment effects (*β*), vertical lines represent 95% confidence intervals and the dashed horizontal line indicates the null effect (*β* = 0). Figure created using Stata 16.1.

The linear mixed-effects models adjusted for covariates did not show significant group differences at baseline for any IMP domain, and also clinical covariates (CP diagnosis, atypical outcome, laterality, MRI severity, and age at baseline) did not significantly influence the outcomes. No evidence of problematic multicollinearity was found (Table 2; Figure 2).

**Table 2:** Motor development measured by the infant motor profile (IMP) and analysed by the Linear mixed-effects models adjusted by the covariates (*n* = 38).

Domain	Variables	*β*	95% CI	*p*-value
Total Score	Assessment time			
	T0	Ref.		
	T1	1.76	0.85 to 2.67	0.002
	T2*	8.83	7.94 to 9.73	<0.001
	T3*	16.42	15.54 to 17.32	<0.001
	Group at T0			
	Infant massage	Ref.		
	CT-R	−2.50	−6.36 to 1.36	0.900
	Outcome (CP)			
	No CP	Ref.		
	CP	−2.61	−7.53 to 2.30	0.971
	Outcome (Atypical)			
	Typical	Ref.		
	Atypical	−5.26	−10.20 to −0.33	0.325
	Laterality			
	Unilateral	Ref.		
	Bilateral	−2.17	−7.00 to 2.66	0.992
	MRI severity			
	Grade 1	Ref.		
	Grade 2	−0.15	−6.16 to 5.86	1.000
	Grade 3	−5.09	−11.96 to 1.77	0.797
	Age			
	Age at T0	0.01	−1.09 to 1.13	1.000
	Time effect (Group × Time)			
	T0 × CT-R	Ref.		
	T1 × CT-R*	5.50	4.23 to 6.78	<0.001
	T2 × CT-R*	4.02	2.76 to 5.28	<0.001
	T3 × CT-R	1.83	0.57 to 3.09	0.050
	*R* ^2^	0.90		
Variation	Assessment time			
	T0	Ref.		
	T1*	5.80	4.32 to 7.30	<0.001
	T2*	12.86	11.40 to 14.33	<0.001
	T3*	16.809	15.34 to 18.27	<0.001
	Group at T0			
	Infant Massage	Ref.		
	CT-R	−1.85	−7.07 to 3.36	0.999
	Outcome (CP)			
	No CP	Ref.		
	CP	−4.35	−10.92 to 2.22	0.886
	Outcome (Atypical)			
	Typical	Ref.		
	Atypical	−7.30	−13.90 to −0.71	0.277
	Laterality			
	Unilateral	Ref.		
	Bilateral	−4.29	−10.74 to 2.15	0.882
	MRI severity			
	Grade 1	Ref.		
	Grade 2	4.29	−3.73 to 12.32	0.970
	Grade 3	−2.32	−11.50 to 6.86	1.000
	Age			
	Age at T0	−0.26	−1.74 to 1.22	1.000
	Time effect (Group × Time)			
	T0 × CT-R	Ref.		
	T1 × CT-R^*^	6.32	4.24 to 8.42	<0.001
	T2 × CT-R^*^	5.50	3.43 to 7.57	<0.001
	T3 × CT-R	1.93	−0.14 to 4.01	0.512
	*R* ^2^	0.82		
Symmetry	Assessment time			
	T0	Ref.		
	T1	3.08	1.45 to 4.71	0.003
	T2^*^	10.23	8.64 to 11.84	<0.001
	T3^*^	10.30	8.71 to 11.90	<0.001
	Group at T0			
	Infant Massage	Ref.		
	CT-R	−2.21	−8.08 to 3.66	0.998
	Outcome (CP)			
	No CP	Ref.		
	CP	−6.60	−14.01 to 0.81	0.580
	Outcome (Atypical)			
	Typical	Ref.		
	Atypical	−2.58	−10.02 to 4.85	0.999
	Laterality			
	Unilateral	Ref.		
	Bilateral	0.20	−7.06 to 7.48	1.000
	MRI severity			
	Grade 1	Ref.		
	Grade 2	−4.20	−13.25 to 4.84	0.990
	Grade 3	−5.89	−16.25 to 4.46	0.955
	Age			
	Age at T0	0.70	−0.97 to 2.37	0.996
	Time effect (Group × Time)			
	T0 × CT-R	Ref.		
	T1 × CT-R*	6.07	3.79 to 8.36	< 0.001
	T2 × CT-R	0.95	−1.31 to 3.22	0.995
	T3 × CT-R	0.87	−1.38 to 3.14	0.998
	*R* ^2^	0.74		
Fluency	Assessment time			
	T0	Ref.		
	T1	−0.77	−2.50 to 0.95	0.992
	T2*	3.77	2.08 to 5.46	<0.001
	T3*	11.40	9.71 to 13.10	<0.001
	Group at T0			
	Infant Massage	Ref.		
	CT-R	−3.69	−7.90 to 0.51	0.600
	Outcome (CP)			
	No CP	Ref.		
	CP	5.45	0.32 to 10.59	0.330
	Outcome (Atypical)			
	Typical	Ref.		
	Atypical	−3.43	−8.58 to 1.71	0.880
	Laterality			
	Unilateral	Ref.		
	Bilateral	2.34	−2.69 to 7.38	0.989
	MRI severity			
	Grade 1	Ref.		
	Grade 2	−4.60	−10.87 to 1.65	0.803
	Grade 3	−10.13	−17.30 to −2.97	0.062
	Age			
	Age at T0	0.14	−1.01 to 1.29	1.000
	Time effect (Group × Time)			
	T0 × CT-R	Ref.		
	T1 × CT-R	4.50	2.08 to 6.93	0.003
	T2 × CT-R	4.56	2.16 to 6.96	0.002
	T3 × CT-R	1.31	−1.08 to 3.71	0.964
	*R* ^2^	0.60		
Performance	Assessment time			
	T0	Ref.		
	T1^*^	6.43	4.88 to 7.98	<0.001
	T2^*^	16.92	15.40 to 18.45	<0.001
	T3^*^	30.82	29.30 to 32.35	<0.001
	Group at T0			
	Infant Massage	Ref.		
	CT-R	−1.69	−7.40 to 4.01	1.000
	Outcome (CP)			
	No CP	Ref.		
	CP	−8.76	−15.97 to −1.56	0.173
	Outcome (Atypical)			
	Typical	Ref.		
	Atypical	−7.16	−14.39 to 0.07	0.429
	Laterality			
	Unilateral	Ref.		
	Bilateral	−8.63	−15.70 to −1.56	0.169
	MRI severity			
	Grade 1	Ref.		
	Grade 2	7.48	−1.31 to 16.28	0.642
	Grade 3	1.95	−8.12 to 12.02	1.000
	Age			
	Age at T0	0.25	−1.37 to 1.88	1.000
	Time effect (Group × Time)			
	T0 × CT-R	Ref.		
	T1 × CT-R^*^	5.65	3.49 to 7.82	<0.001
	T2 × CT-R	1.66	−0.49 to 3.81	0.757
	T3 × CT-R	2.93	0.78 to 5.08	0.082
	*R* ^2^	0.89		

^*^Bonferroni-adjusted *p*-values <0.01.

Ref, reference variable; CT-R, CareToy-Revised; T0, baseline assessment before the start of the intervention; T1, end of the 8-week intervention period (primary endpoint); T2, follow-up 2 months after T1; T3, follow-up at 18 months of age; *β*, fixed-effect coefficients from linear mixed-effects models for Infant Motor Profile (IMP) raw scores; 95% CI, 95% confidence interval. *R*^2^, the proportion of variance in IMP scores explained by the model.

The CT-R and IM score and delta score in IMP were significantly different from T1 to T3 (Supplementary Figure S1). For the IMP total score, a significant effect of assessment time was observed, with increases at T2 (*β* = 8.83, SE = 0.45, *t* = 19.43, 95% CI = 7.94 to 9.73, *p* < 0.001), and T3 (*β* = 16.42, SE = 0.45, *t* = 36.14, 95% CI = 15.54 to 17.32, *p* < 0.001). Significant time × group interactions were found at T1 (*β* = 5.5, SE = 0.649, *t* = 8.48, 95% CI = 4.23 to 6.78, *p* < 0.001), T2 (*β* = 4.02, SE = 0.64, *t* = 6.25, 95% CI = 2.76 to 5.28, *p* < 0.001). The model explained a high proportion of variance (*R*^2^ = 0.90).

For the IMP variation domain, assessment time had a significant effect at T1 (*β* = 5.80, SE = 0.761, *t* = 7.63, 95% CI = 4.32 to 7.30, *p* < 0.001), T2 (*β* = 12.86, SE = 0.74, *t* = 17.20, 95% CI = 11.40 to 14.33, *p* < 0.001), and T3 (*β* = 16.80, SE = 0.74, *t* = 22.48, 95% CI = 15.34 to 18.27, *p* < 0.001). Significant time × group interactions were observed at T1 (*β* = 6.32, SE = 1.06, *t* = 5.93, 95% CI = 4.24 to 8.42, *p* < 0.001) and T2 (*β* = 5.50, SE = 1.05, *t* = 5.20, 95% CI = 3.43 to 7.57, *p* < 0.001). The explained variance was high (*R*^2^ = 0.82).

For IMP symmetry, significant effects of time were detected at T2 (*β* = 10.23, SE = 0.81, *t* = 12.54, 95% CI = 8.64 to 11.84, *p* < 0.001), and T3 (*β* = 10.30, SE = 0.81, *t* = 12.62, 95% CI = 8.71 to 11.90, *p* < 0.001). A significant time × group interaction was observed at T1 (*β* = 6.07, SE = 1.16, *t* = 5.21, 95% CI = 3.79 to 8.36, *p* < 0.001). The model showed good fit (*R*^2^ = 0.74).

For IMP fluency, significant time effects were observed at T2 (*β* = 3.77, SE = 0.86, *t* = 4.362, 95% CI = 2.08 to 5.46, *p* < 0.001) and T3 (*β* = 11.40, SE = 0.86, *t* = 13.18, 95% CI = 9.71 to 13.10, *p* < 0.001). A trend toward significance regarding time × group interactions was present at T2 (*β* = 4.56, SE = 1.22, *t* = 3.73, 95% CI = 2.16 to 6.96, *p* = 0.002). The explained variance was moderate (*R*^2^ = 0.60).

For IMP performance, assessment time showed significant effects at T1 (*β* = 6.43, SE = 0.79, *t* = 8.14, 95% CI = 4.88 to 7.98, *p* < 0.001), T2 (*β* = 16.92, SE = 0.77, *t* = 21.80, 95% CI = 15.40 to 18.45, *p* < 0.001), and T3 (*β* = 30.82, SE = 0.77, *t* = 39.719, 95% CI = 29.30 to 32.35, *p* < 0.001). A significant time × group interaction was observed at T1 (*β* = 5.65, SE = 1.10, *t* = 5.108, 95% CI = 3.49 to 7.82, *p* < 0.001). The model explained a large proportion of variance (*R*^2^ = 0.89) (Supplementary Table S2). Exploratory post-hoc analysis showed the highest increases in IMP total score from T0 to T3 (estimate = −18.26, *p* < 0.001), performance (T0 to T3: estimate = −33.76, *p* < 0.001) and variation (T0 to T3: estimate = −18.75, *p* < 0.001) within the CT-R group. Within the IM group, post-hoc comparisons showed the highest increases in IMP total score from T0 to T3 (estimate = −16.43, *p* < 0.001), performance (T0 to T3: estimate = −30.82, *p* < 0.001) and variation (T0 to T3: estimate = −16.81, *p* < 0.001).

## 4 Discussion

This randomized clinical trial investigated the effects of CT-R, a home-based early technological intervention, on the neurodevelopment of infants at high risk of CP, compared with IM, an established EI approach. The main findings indicate that both interventions were associated with developmental improvements over time, but CT-R produced significantly greater gains in early motor development, particularly in the IMP total score and key domains such as variation, performance, and symmetry. It is important to highlight that the IMP measures motor development not only quantitatively (performance), but above all qualitatively (variation, symmetry, fluency, and adaptability), providing information on the infant’s movement repertoire and motor behaviour organization, without focusing only on milestones (6). According to the Neuronal Group Selection Theory, infants with an early lesion of the brain may show reduced variation; therefore, changes in the qualitative domains of the IMP are clinically relevant and may reflect central nervous system functions and structures (50). In addition, it is a playful and minimally invasive assessment where the assessor needs to touch the infant as minimal as possible, so that the infants can perform their development in an ecological way (6).

The early motor development improvement observed in both groups is consistent with the expected developmental trajectory during infancy and with previous evidence showing that EI, regardless of modality, supports motor and neurodevelopment in high-risk populations. However, the stronger effects detected in the CT-R group suggest that an intensive, goal-directed and enriched intervention may provide additional benefits compared with traditional parent-delivered approaches such as infant massage.

One of the main differences between the two approaches is technology. In the rehabilitation landscape, the adoption of various technologies has revolutionized patient care, enabled quantitative monitoring and supporting clinical decision-making. Most importantly, it has facilitated the use of telerehabilitation, allowing clinical rehabilitation to be integrated directly into patients’ homes while ensuring remote monitoring and continuous adaptation of treatment programs to patient’s changing needs (51).

Within this evolving framework, EI represents a particularly relevant field for technological innovation, given the need for timely, intensive, and family-centered interventions during critical windows of neuroplasticity.

In this context, the present study provides evidence supporting the integration of technology into EI pathways, demonstrating how remotely supervised, home-based intervention can be implemented while maintaining individualized goal setting. In this perspective, technology should not be interpreted as a replacement for parental interaction, but rather as a tool that can support parents in providing a richer and more structured motor experience for their infants. Devices such as CT-R can contribute to a more personalized rehabilitation approach by enabling remote supervision and continuous updating of treatment goals. This creates concrete opportunities to support EI by bridging the home environment and clinical care (52). In addition, approaches such as CT-R may allow infants to remain in a familiar and ecological setting, in which families can choose the most appropriate moments for training (e.g., considering feeding and sleeping times), facilitating the integration of intervention within daily life rather than an external activity.

In this RCT the effect of CT-R was evident at the end of the 8-week intervention at T1, and it remained significant at the 2-month follow-up at T2 for the IMP total score and variation domains. It is important to note that the total dose was similar across groups, and motor outcome at T1 was not significantly correlated with intervention dose. Thus, it confirms that the CT-R effect at T1 was not related to doing more training. Moreover, although both groups received two EI models with proven effectiveness, the group that also received CT-R showed greater improvement. This highlights the specific contribution of the technology-based intervention in enhancing parents’ ability to promote early development. In addition, the results of this study need to be framed within the context of standard care; in fact, the effects of EI must be considered as additive to an already high-quality developmental support framework, characterized by high standards, periodic assessments, and parent coaching. The significant impact on early developmental promotion confirms that it is possible to further enrich the environment to better support infants’ growth.

Consistent with previously published data, the effect of CT-R training was mainly reflected by the IMP scores (primary outcome measure) and especially by the variation domain. This finding was not unexpected, since CT-R was developed with the stated aim of enriching the variation of the infant motor repertoire offering diverse motor activities with different degrees of complexity. From a neurodevelopmental perspective, increased variation in motor behaviour may reflect a richer motor repertoire and a more flexible selection of motor strategies, processes that are considered fundamental for the maturation of motor networks during early brain development. In fact, variation of the infant motor behaviour is increasingly considered as a faithful expression of the complexity of brain connectivity and a solid predictor of poor motor performance in infants with brain injury (53, 54).

Previous studies have reported a significant effect of both IM and CT in improving visual acuity of premature infants without brain injury (17, 55). In our study, visual acuity improved throughout the study with no significant differences between the two groups. At 18 months, both groups showed normal visual acuity for age confirming that the two interventions have a comparable effect over visual functions (34). Since the presence of retinopathy of premature was considered an exclusion criterium for the study, it is possible that we selected those infants whose visual function was already better preserved. Also, in infants with cerebral visual impairment due to early brain injury, acuity is only one of the many aspects of vision which can be affected (56). The mere assessment of visual acuity with TAC may have prevented us from detecting more subtle and complex impairments of visual function.

Previous data demonstrated a small positive effect of EI on cognitive outcome (57). In our study, cognitive measures at 18 months were comparable between the two groups. This is probably explained by the relatively limited duration of the intervention period (8 weeks) which may have prevented a significant improvement of cognitive performance. Future studies will try to elucidate whether a longer duration of the intervention could lead to an improvement in cognitive skills.

The current study adds to a growing list of home-based EI programs based on the concept of the enriched environment (17, 45, 58–60). In general, these programs are based on a parent-delivered intervention which is periodically supervised and updated by professionals. These EI are hardly standardizable since frequency, dose of the intervention and compliance of caregivers are hardly measurable. Unlike most of these studies, the intervention with CT-R was entirely performed at home, as the IM, but thanks to its technological architecture, it enabled continuous monitoring, remote adjustment of training parameters and personalized feedback, potentially supporting treatment fidelity and optimizing dosage. This may explain why treatment dose alone was not significantly correlated with outcomes, suggesting that the quality and specificity of practice, rather than quantity alone, are key determinants of effectiveness.

Part of this study (February 2020 – June 2020) was conducted during the global COVID-19 pandemic. During the sanitary emergency, access to non-essential rehabilitation services was severely limited with most of the outpatient rehabilitative activities suspended throughout Europe (61). Lockdown and social distancing measures have greatly narrowed the possibility of delivering physical rehabilitation with a negative impact especially on infants and children with special needs (62). The use of a telerehabilitation platform as CT-R has allowed the participants to proceed with the scheduled activities, without affecting the quality of the intervention and maintaining close contact with the rehabilitation professionals who telemonitored the program.

Considering the overall effects of the training on early motor development, the persistence of between-group differences at follow-up further supports the hypothesis that early enriched motor experiences can influence longer-term developmental trajectories. Although the magnitude of the interaction decreased over time, CT-R maintained advantages in several IMP domains, indicating that early gains may be translated into more efficient motor learning processes. These findings align with literature highlighting the importance of active, exploratory interventions that promote self-initiated movement and problem solving in infants at neurological risk (10).

In our study, the great majority of the participants had a moderate to severe brain injury suggesting that our study recruited sample may be more severely affected than a representative CP population sample (63). This can be explained by the fact that one of the three participating NICUs (Meyer Children Hospital, Florence, Italy) was as a referral center for neonatal neurologic disorders admitting the most critical patients from all over the region. This may have affected the representativeness of the study sample. Nevertheless, it is important to mention that in the linear mixed-effects models adjusted for clinical covariates (including MRI severity), the treatment effect (Group × Time) remained significant at early time points, while MRI severity and the other covariates did not significantly influence the IMP outcomes (Table 2). Therefore, our findings support the use of CT-R in a clinically complex population; however, larger studies are needed to specifically investigate whether responsiveness to CT-R differs across brain injury severity levels.

### 4.1 Strengths and limitations

A strength of this study is its evaluator-blinded randomized design, comparing CT-R with an active intervention of similar dose minimizing potential biases, such as assessor bias. Secondly, the home-based format of CT-R has the potential to increase access to EI by limiting the burden of repeated travel to specialized centers and by supporting treatment delivery within daily family routines. Delivering training at home helps families take advantage of a sensitive developmental window within the first 1,000 days, when infants are more responsive to environmental inputs and neuroplasticity is high. However, the study has limitations. First, the intervention lasted 8 weeks; this duration may be insufficient to produce measurable differences in domains such as cognition, and longer programs may be needed. Second, part of the trial occurred during the COVID-19 pandemic. Although the telerehabilitation approach made it possible to continue delivering treatment to families, the pandemic may also have influenced family routines and daily organization. Third, participation in standard care alongside the experimental interventions may have introduced variability that could not be fully controlled. Moreover, the relatively small sample size and the use of unstratified randomization represent additional limitations, as they may have increased the risk of chance baseline imbalances. However, IMP assessments were scored after trial completion by blinded assessors and were therefore not available for stratification. Finally, when it comes to external generalizability, as previous discussed, technology-based home EI access may not be uniform for all families. Limited availability of suitable devices, unstable internet connection, and lack of familiarity with technology may have reduced accessibility. It is also important to highlight that the absence of significant Group × Time interactions for most IMP domains at T3 does not allow conclusions about long-term effects of CT-R on developmental trajectories, as the observed findings may also reflect a gradual catch-up of the IM group through maturation and continued standard care.

## 5 Conclusion

Our study suggests that an 8-week EI program with CT-R has a positive effect on early development of motor function in infants with brain injury at high-risk of CP, with greater improvements compared with IM during a sensitive window of neuroplasticity and supporting the importance of a family-centered framework. CT-R integrates key features recommended for effective EI, including early start, high intensity, goal-directed activities, an enriched environment, and a family-centered approach. As far as we know, CT-R is one of the first technology-based EI specifically designed to be delivered at home and implemented by parents, while being remotely supervised and adapted by rehabilitation professionals, potentially increasing access to EI for families who live far from specialized centers or have limited access to specialized healthcare services. Although further large-scale studies are needed to confirm these findings and evaluate long-term functional and cost-effectiveness outcomes, CT-R may represent an innovative and scalable approach to enhance early neurodevelopment and potentially mitigate the severity of motor impairment in infants at high risk for CP.

## Data Availability

The raw data supporting the conclusions of this article will be made available by the authors, without undue reservation.
