# Supplementary material for: Home based early intervention with CareToy-Revised in infants with early brain injury: a randomized controlled trial
Source: Front Med (Lausanne). 2026 Aug 12;13:1830574. doi: 10.3389/fmed.2026.1830574 (PMC13506885; doi:10.3389/fmed.2026.1830574)
Supplement: Supplementary file 1 [file Supplementary_file_1.docx]

Supplementary Material

# 1 Figure 1

| **Column A** | **Column B** |
| --- | --- |
|  |  |
|  |  |
|  |  |
|  |  |
|  |  |

*Figure 1. Raw Scores and Change Scores Across Time Intervals by Group (0 = Infant Massage; 1 = CareToy-R)*

*Note: Column A shows the figures regarding the raw score of Infant Motor Profile (IMP) domains. Boxplots show change in raw scores in the IMP domains for each time assessment and study group (0 = Infant Massage; 1 = CareToy-R), interquartile range, and data dispersion. Horizontal lines and red diamonds represent medians and means, respectively. Black dots represent outliers. Column B shows the figures regarding the change scores (Δ) of the IMP domains. Boxplots display the delta between each assessment time (for instance, T1-T0, T2-T0, T2-T1, T3-T0, T3-T1, T3-T2), according to the study group (0 = Infant Massage; 1 = CareToy-R). They are illustrating interquartile ranges and data dispersion. Horizontal lines indicate medians, and red diamonds represent means. Black dots represent outliers. 1,CareToy-Revised; 0, infant massage;T0, baseline assessment before the start of the intervention; T1, end of the 8-week intervention period (primary endpoint); T2, follow-up 2 months after T1; T3, follow-up at 18 months of age.*

# 2 Table 1

*Table 1. Significant post-hoc contrasts for Infant Motor Profile scores (within- and between-group comparisons)*

| ***IMP total score*** | | | | | | | |
| --- | --- | --- | --- | --- | --- | --- | --- |
| **Group** | **Contrast** | **Estimate** | **SE** | **df** | **t.ratio** | **p.value** | **95% CI** |
|  | T0 group0 – T1 group0 | -1.76003 | 0.46283 | 862.32074 | -3.80278 | 0.00381 | -0.78867 to -0.23711 |
|  | T0 group0 – T2 group0 | -8.83208 | 0.45452 | 862.00003 | -19.43156 | 0.0 | -2.87074 to -2.27680 |
|  | T0 group0 – T3 group0 | -16.42868 | 0.45452 | 862.00003 | -36.14491 | 0.0 | -5.14270 to -4.43232 |
|  | T1 group0 – T2 group0 | -7.07206 | 0.46283 | 862.32074 | -15.28017 | 0.0 | -2.35300 to -1.76876 |
|  | T1 group0 – T3 group0 | -14.66865 | 0.46283 | 862.32074 | -31.69369 | 0.0 | -4.61815 to -3.93109 |
|  | T2 group0 – T3 group0 | -7.5966 | 0.45452 | 862.00003 | -16.71336 | 0.0 | -2.50387 to -1.92361 |
| IMP total score -Within the group (Group 1 = CT-R) | T0 group1 – T1 group1 | -7.26575 | 0.45452 | 862.00003 | -15.98546 | 0.0 | -2.40580 to -1.82885 |
|  | T0 group1 – T2 group1 | -12.8522 | 0.45452 | 862.00003 | -28.27627 | 0.0 | -4.07003 to -3.42054 |
|  | T0 group1 – T3 group1 | -18.2584 | 0.45452 | 862.00003 | -40.17049 | 0.0 | -5.69320 to -4.94823 |
|  | T1 group1 – T2 group1 | -5.58645 | 0.45452 | 862.00003 | -12.29081 | 0.0 | -1.90894 to -1.34698 |
|  | T1 group1 – T3 group1 | -10.99264 | 0.45452 | 862.00003 | -24.18503 | 0.0 | -3.51433 to -2.89244 |
|  | T2 group1 – T3 group1 | -5.40619 | 0.45452 | 862.00003 | -11.89422 | 0.0 | -1.85571 to -1.29515 |
| ***Variation*** | | | | | | | |
| **Group** | **Contrast** | **Estimate** | **SE** | **df** | **t.ratio** | **p.value** | **95% CI** |
| Variation -Within the group (Group 0 = Infant Massage) | T0 group0 – T1 group0 | -5.80832 | 0.76106 | 862.48223 | -7.63192 | 0.0 | -1.30803 to -0.75056 |
|  | T0 group0 – T2 group0 | -12.86106 | 0.74743 | 862.00006 | -17.20695 | 0.0 | -2.57003 to -1.98820 |
|  | T0 group0 – T3 group0 | -16.80874 | 0.74743 | 862.00006 | -22.4886 | 0.0 | -3.28394 to -2.67343 |
|  | T1 group0 – T2 group0 | -7.05274 | 0.76106 | 862.48223 | -9.26703 | 0.0 | -1.53064 to -0.96900 |
|  | T1 group0 – T3 group0 | -11.00042 | 0.76106 | 862.48223 | -14.45415 | 0.0 | -2.23933 to -1.65946 |
|  | T2 group0 – T3 group0 | -3.94769 | 0.74743 | 862.00006 | -5.28165 | 0.0 | -0.97105 to -0.42809 |
| Variation -Within the group (Group 1 = CT-R) | T0 group1 – T1 group1 | -12.13757 | 0.74743 | 862.00006 | -16.23899 | 0.0 | -2.43959 to -1.86223 |
|  | T0 group1 – T2 group1 | -18.36368 | 0.74743 | 862.00006 | -24.56897 | 0.0 | -3.56601 to -2.94247 |
|  | T0 group1 – T3 group1 | -18.74507 | 0.74743 | 862.00006 | -25.07923 | 0.0 | -3.63524 to -3.00841 |
|  | T1 group1 – T2 group1 | -6.22611 | 0.74743 | 862.00006 | -8.32998 | 0.0 | -1.37795 to -0.82872 |
|  | T1 group1 – T3 group1 | -6.6075 | 0.74743 | 862.00006 | -8.84024 | 0.0 | -1.44618 to -0.89566 |
| ***Symmetry*** | | | | | | | |
| **Group** | **Contrast** | **Estimate** | **SE** | **df** | **t.ratio** | **p.value** | **95% CI** |
| Symmetry -Within the group (Group 0 = Infant Massage) | T0 group0 – T1 group0 | -3.07955 | 0.83114 | 862.4528 | -3.70523 | 0.00549 | -0.77513 to -0.22431 |
|  | T0 group0 – T2 group0 | -10.2381 | 0.81625 | 862.00005 | -12.54278 | 0.0 | -1.94243 to -1.38024 |
|  | T0 group0 – T3 group0 | -10.30284 | 0.81625 | 862.00005 | -12.6221 | 0.0 | -1.95308 to -1.39060 |
|  | T1 group0 – T2 group0 | -7.15854 | 0.83114 | 862.4528 | -8.61295 | 0.0 | -1.44163 to -0.88160 |
|  | T1 group0 – T3 group0 | -7.22329 | 0.83114 | 862.4528 | -8.69085 | 0.0 | -1.45223 to -0.89201 |
| Symmetry -Within the group (Group 1 = CT-R) | T0 group1 – T1 group1 | -9.15426 | 0.81625 | 862.00005 | -11.21497 | 0.0 | -1.76429 to -1.20662 |
|  | T0 group1 – T2 group1 | -11.19084 | 0.81625 | 862.00005 | -13.70999 | 0.0 | -2.09928 to -1.53259 |
|  | T0 group1 – T3 group1 | -11.18225 | 0.81625 | 862.00005 | -13.69947 | 0.0 | -2.09787 to -1.53121 |
| ***Fluency*** | | | | | | | |
| **Group** | **Contrast** | **Estimate** | **SE** | **df** | **t.ratio** | **p.value** | **95% CI** |
| Fluency -Within the group (Group 0 = Infant Massage) | T0 group0 – T2 group0 | -3.77193 | 0.86479 | 862.00029 | -4.36167 | 0.00038 | -0.84739 to -0.30804 |
|  | T0 group0 – T3 group0 | -11.40351 | 0.86479 | 862.00029 | -13.18643 | 0.0 | -2.02763 to -1.46554 |
|  | T1 group0 – T2 group0 | -4.54477 | 0.88043 | 863.03511 | -5.16202 | 1e-05 | -0.97122 to -0.42095 |
|  | T1 group0 – T3 group0 | -12.17635 | 0.88043 | 863.03511 | -13.83008 | 0.0 | -2.15235 to -1.57756 |
|  | T2 group0 – T3 group0 | -7.63158 | 0.86479 | 862.00029 | -8.82477 | 0.0 | -1.44292 to -0.89482 |
| Fluency -Within the group (Group 1 = CT-R) | T0 group1 – T1 group1 | -3.72789 | 0.86479 | 862.00029 | -4.31075 | 0.00048 | -0.84068 to -0.30126 |
|  | T0 group1 – T2 group1 | -8.33333 | 0.86479 | 862.00029 | -9.63624 | 0.0 | -1.55158 to -1.00113 |
|  | T0 group1 – T3 group1 | -12.71912 | 0.86479 | 862.00029 | -14.70774 | 0.0 | -2.23224 to -1.66394 |
|  | T1 group1 – T2 group1 | -4.60544 | 0.86479 | 862.00029 | -5.32549 | 0.0 | -0.97582 to -0.43494 |
|  | T1 group1 – T3 group1 | -8.99123 | 0.86479 | 862.00029 | -10.397 | 0.0 | -1.65346 to -1.10077 |
|  | T2 group1 – T3 group1 | -4.38579 | 0.86479 | 862.00029 | -5.0715 | 1e-05 | -0.94198 to -0.40149 |
| ***Performance*** | | | | | | | |
| **Group** | **Contrast** | **Estimate** | **SE** | **df** | **t.ratio** | **p.value** | **95% CI** |
| Performance -Within the group (Group 0 = Infant Massage) | T0 group0 – T1 group0 | -6.43239 | 0.79017 | 862.43322 | -8.14047 | 0.0 | -1.37736 to -0.81844 |
|  | T0 group0 – T2 group0 | -16.92455 | 0.77602 | 862.00005 | -21.8094 | 0.0 | -3.19209 to -2.58537 |
|  | T0 group0 – T3 group0 | -30.82305 | 0.77602 | 862.00005 | -39.71936 | 0.0 | -5.63107 to -4.89084 |
|  | T1 group0 – T2 group0 | -10.49216 | 0.79017 | 862.43322 | -13.27829 | 0.0 | -2.07850 to -1.50316 |
|  | T1 group0 – T3 group0 | -24.39066 | 0.79017 | 862.43322 | -30.86744 | 0.0 | -4.50306 to -3.82306 |
|  | T2 group0 – T3 group0 | -13.8985 | 0.77602 | 862.00005 | -17.90995 | 0.0 | -2.66499 to -2.07947 |
| Performance -Within the group (Group 1 = CT-R) | T0 group1 – T1 group1 | -12.08919 | 0.77602 | 862.00005 | -15.57844 | 0.0 | -2.35072 to -1.77611 |
|  | T0 group1 – T2 group1 | -18.58495 | 0.77602 | 862.00005 | -23.94904 | 0.0 | -3.48204 to -2.86221 |
|  | T0 group1 – T3 group1 | -33.75527 | 0.77602 | 862.00005 | -43.49788 | 0.0 | -6.14854 to -5.37433 |
|  | T1 group1 – T2 group1 | -6.49576 | 0.77602 | 862.00005 | -8.3706 | 0.0 | -1.38348 to -0.83394 |
|  | T1 group1 – T3 group1 | -21.66607 | 0.77602 | 862.00005 | -27.91945 | 0.0 | -4.02121 to -3.37483 |
|  | T2 group1 – T3 group1 | -15.17031 | 0.77602 | 862.00005 | -19.54885 | 0.0 | -2.88635 to -2.29226 |

*Note: IMP, Infant Motor Profile; CT-R, CareToy–Revised; IM, Infant Massage; T0, baseline assessment before the start of the intervention; T1, end of the 8-week intervention period (primary endpoint); T2, follow-up 2 months after T1; T3, follow-up at 18 months of age; SE, standard error; df, degrees of freedom; CI, confidence interval.*
